# Challenges and Insights in Patch-Clamp Studies: From Cell-Attached to Whole-Cell Configurations

**DOI:** 10.3390/cimb48020137

**Published:** 2026-01-27

**Authors:** Sheng-Nan Wu, Ya-Jean Wang, Rasa Liutkevičienė

**Affiliations:** 1 Department of Research and Education, An Nan Hospital, China Medical University, No. 66, Section 2, Changhe Road, An Nan District, Tainan 70965, Taiwan; 2 Department of Senior Service Industry Management, Minghsin University of Science and Technology, 1 Xinxing Road, Xinfeng Township, Hsinchu 304001, Taiwan; yawang@must.edu.tw; 3 Laboratory of Ophthalmology, Institute of Neuroscience, Lithuanian University of Health Sciences, Kaunas 50106, Lithuania; rasa.liutkeviciene@lsmuni.lt

**Keywords:** patch-clamp technique, whole-cell configuration, cell-attached configuration, action current, ion-channel activity, mitoxantrone, GAL-021, brain slice patch-clamp recording

## Abstract

The patch-clamp technique is widely regarded as the gold standard in cellular electrophysiology and can be applied in several configurations. In the cell-attached (C-A) mode, it enables the recording of single-channel currents, whereas the whole-cell (W-C) mode allows for the measurement of macroscopic currents, representing the collective activity of many channels. When the recording configuration was switched from C-A to W-C on the same cell, the current amplitude increased dramatically, while action currents (ACs) were completely abolished, indicating a profound alteration in the cell’s electrophysiological response under the new setup. In excitable cells, the occurrence of ACs, representing propagated action potentials, can interfere with C-A single-channel recordings. To address this, a high-K^+^ solution is typically applied to the bath to suppress the ACs. The inwardly rectifying K^+^ (Kir), ATP-sensitive K^+^ (K_ATP_) and large-conductance Ca^2+^-activated K^+^ (BK_Ca_) channels are crucial members of the K^+^ channel family that facilitate the efflux of K^+^ ions, driven by the K^+^ electrochemical gradient. These channels are primarily distinguished by their rectification properties and gating kinetics. For instance, K_ATP_ channels exhibit a bursting kinetic pattern with inward rectifying property, while BK_Ca_ channels display strong outward rectification. Mitoxantrone, which belongs to a class of drugs called anthracenediones, can suppress the activity of Kir channels in differentiated RAW 264.7 cells, with no change in single-channel conductance. The respiratory stimulator GAL-021 acts as a BK_Ca_ channel inhibitor, and it suppresses channel activity and shifts the activation curve to the right, suggesting a voltage-dependent blockade that stabilizes the channel in a closed state. GAL-021 does not change the single-channel conductance, indicating it is a gating modifier rather than an open-pore blocker. The functional roles of ion channels are fundamentally important. Correspondingly, the field is transitioning to artificial intelligence for automated single-cell patch-clamp experiments, though brain slice recordings still require manual techniques.

## 1. Introduction

**Figure 1:**
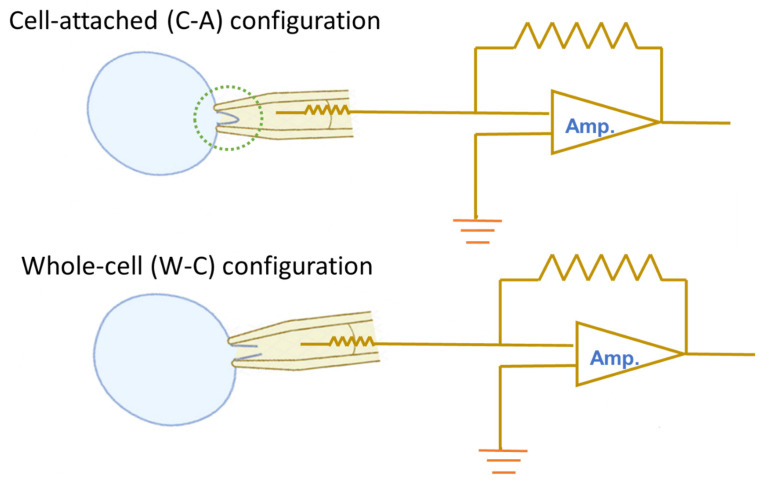
Schematic illustration of cell-attached (C-A, **upper**) and whole-cell (W-C, **lower**) configurations in the patch-clamp recordings. In the C-A mode, the gentle negative pressure applied to the pipette draws the cell membrane tightly against the electrode tip, producing a U-shaped invagination within the pipette (dotted green circle). This high-resistance gigaseal (>1 GΩ) enables measurement of ionic currents across the isolated membrane patch. Transition to the W-C mode occurs when additional suction ruptures the membrane beneath the pipette tip, causing a sudden increase in membrane capacitance while maintaining a tight seal at the rim. This configuration permits recording of ligand-gated and voltage-gated ion currents. On the right of each panel, a simplified amplifier (Amp.) circuit diagram is shown, highlighting signal amplification. Light blue circles indicate the targeted cell. Voltage-clamp and current-clamp recordings can be conducted in both C-A and W-C modes.

The patch-clamp technique is widely recognized as the gold standard in electrophysiology, owing to its unparalleled ability to provide high-resolution insights into the electrical activity of cells and their ion channels. Its strength lies in the versatility of its experimental configurations, which allow researchers to precisely manipulate and control the conditions on both sides of the cell membrane [1]. The commonly used patch-clamp configurations are cell-attached (C-A), inside-out, outside-out, and whole-cell (W-C) modes [1,2]. Of these, the cell-attached (C-A) and whole-cell (W-C) modes are the two most frequently used. Figure 1 is a schematic cartoon that briefly illustrates the main differences between the two configurations. However, their operation often involves specific challenges, such as the direction of ionic flow across the membrane and the associated rectification properties. Rectification in ion channel dynamics describes the phenomenon where ions pass more readily in one direction than in the other, a behavior analogous to that observed in semiconductor devices. This article aims to present essential information and clear key concepts commonly encountered during operation, serving as a useful reference.

## 2. Ionic Currents Evoked by Ramp or Rectangular Pulse Recorded Under Cell-Attached (C-A) and Whole-Cell (W-C) Voltage-Clamp Configurations

**Figure 2:**
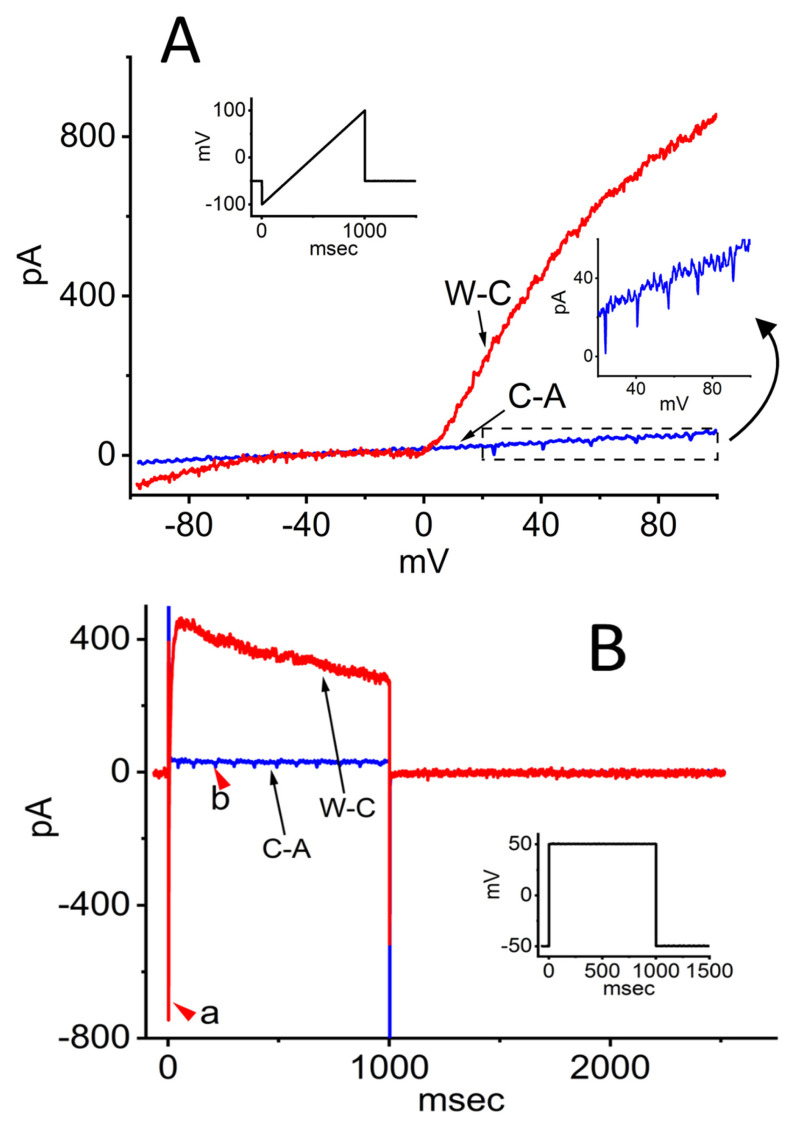
Ionic currents in response to ramp and rectangular pulse stimulation in INS-1 pancreatic β-cells. Cells were bathed in normal Tyrode’s solution containing 1.8 mM CaCl_2_, and recordings were obtained using electrodes filled with a K^+^-based internal solution. Both cell-attached (C-A) and whole-cell (W-C) voltage-clamp recordings were performed in the same cell. Panels (**A**) and (**B**) show representative current traces under C-A and W-C configurations, respectively. Insets illustrate the voltage protocol applied. In (**A**), the lower right inset displays an expanded view of the boxed region, highlighting amplified action currents (ACs). In (**B**), the red arrowhead labeled “a” marks the voltage-gated Na^+^ current (*I*_Na_) recorded in W-C mode, while arrowhead “b” indicates the appearance of ACs in C-A mode. The downward deflections in the inset of (**A**) correspond to the occurrence of ACs.

As illustrated in Figure 2A, during these measurements, INS-1 pancreatic β-cells were maintained in standard Tyrode’s solution, and recordings were obtained using pipettes filled with a K^+^-rich internal solution. INS-1 pancreatic β-cells are a rat insulinoma-derived cell line widely used as a model to study pancreatic β cell physiology, glucose metabolism, and insulin secretion [3,4]. In the C-A configuration, after achieving a gigaohm seal, ramp pulses of 1 s duration evoked only relatively small ionic currents. Notably, a repetitive inward current was observed during the upsloping phase of the ramp, with a threshold potential near −22 mV. The amplitude of this inward current (shown in blue) gradually diminished and broadened as the membrane depolarized.

Upon transition to the W-C configuration following patch rupture, the ramp-evoked currents (shown in red) increased dramatically—approximately 20-fold compared to C-A recordings. In the same cells, membrane resistance between –40 and +40 mV decreased sharply from 2.21 ± 0.03 to 0.089 ± 0.01 GΩ (*n* = 12, *p* < 0.05). Interestingly, the trains of action currents (ACs) evident in C-A mode were abolished under W-C conditions, leaving only a small inward current around 0 mV. Furthermore, both inwardly rectifying K^+^ currents of modest amplitude and prominent outwardly rectifying K^+^ currents were detected in response to the ramp protocol.

To better characterize the biophysical properties of ionic currents in INS-1 cells, command voltage was switched to a rectangular pulse protocol. As shown in Figure 2B, under the C-A mode, when the cell was held at –50 mV and depolarized to +50 mV for 1 s, a small outward current (blue trace) was observed together with a train of inward currents (red arrowhead, labeled b). These inward currents exhibited spike-frequency adaptation and are referred to as ACs, corresponding to the generation of propagated action potentials (APs) [5,6,7,8,9]. Spike-frequency adaptation in excitable cells refers to the gradual reduction in firing rate during sustained constant current injection, caused by intrinsic ionic mechanisms that limit excitability over time. Notably, once the patch membrane was ruptured, the current amplitude was increased substantially. Transitioning from C-A to W-C mode resulted in a marked reduction in membrane resistance during depolarization from –50 to +50 mV, decreasing from 3.48 ± 0.09 to 0.35 ± 0.02 GΩ (*n* = 12, *p* < 0.05). In the same cells, both a transient voltage-gated Na^+^ current, characterized by rapid activation and inactivation (red arrowhead a), and a delayed-rectifier K^+^ current with slow inactivation were simultaneously recorded. However, while the ACs were observable in the C-A configuration, they were no longer discernible upon transition to the W-C mode. Overall, under these experimental conditions, approximately 70% of INS-1 cells exhibited repetitive AC firing.

## 3. Measurement of Resting Membrane Potential Under C-A Mode

**Figure 3:**
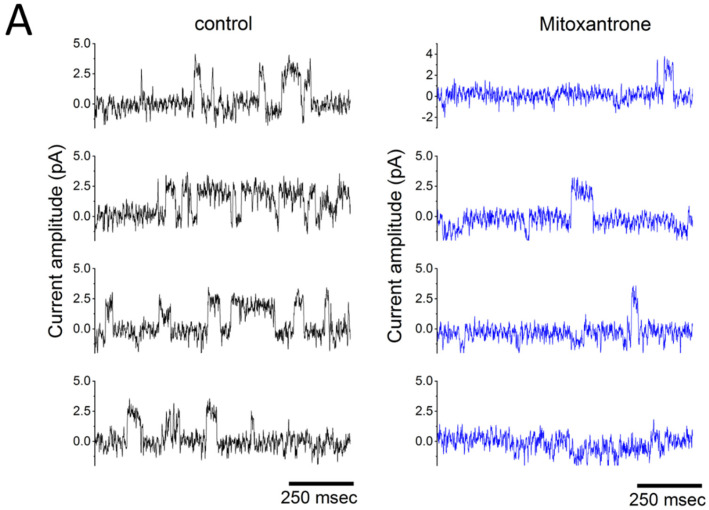

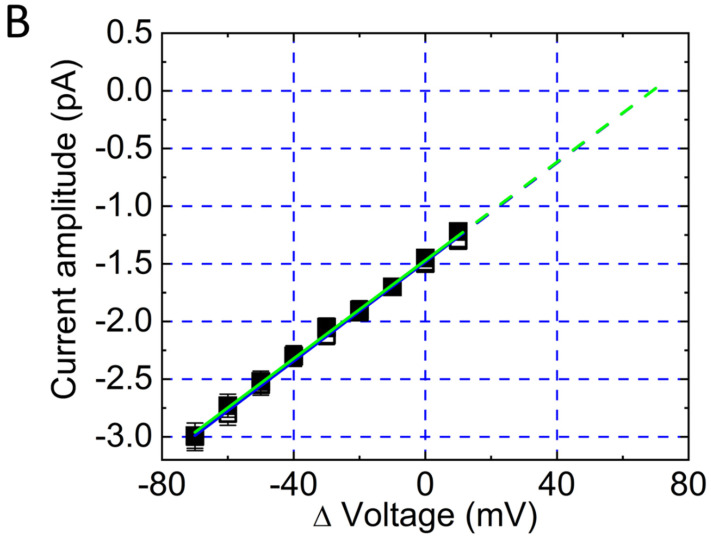
Mitoxantrone inhibits inwardly rectifying K^+^ (Kir) channel activity in lipopolysaccharide-treated RAW 264.7 osteoclast precursor cells. Experiments were performed in cells bathed in normal Tyrode’s solution containing 1.8 mM CaCl_2_. Single-channel recordings were obtained in the cell-attached (C-A) configuration using pipettes filled with or without mitoxantrone (10 μM). The attached cell was held at −50 mV relative to the bath (amplifier holding potential set to +50 mV, corresponding to a membrane potential of approximate −120 mV relative resting; patch potential = +120 mV). (**A**) Representative current traces recorded in the absence (**left**) and presence (**right**) of 10 μM mitoxantrone. Channel openings are displayed as upward deflections when viewed from the pipette electrode; referenced to the patch membrane, openings would appear as downward deflections (negative values). Analysis of single-channel current histograms revealed no difference in current amplitude between control and mitoxantrone-treated cells. (**B**) Averaged current-voltage (I-V) relationships of single Kir channel currents under control conditions (black open squares) and during exposure to 10 μM mitoxantrone (black filled squares). Single-channel amplitudes (negative values from the cell’s perspective) was plotted against Δvoltage relative to the bath. For example, when Δvoltage = 0 or +20 mV, the pipette potential was set at 0 or −20 mV, corresponding to membrane potentials of −70 or −50 mV, respectively, and patch potential of +70 or +50 mV, respectively. Kir-channel openings thus appear as upward deflections from the pipette perspective. The reversal potential (green dashed line) occurs at approximately +70 mV. Importantly, mitoxantrone did not alter single-channel conductance. This figure is adapted from Wang et al. [13] and is published under the Creative Commons Attribution (CC BY) license.

Determining the resting membrane potential can be difficult to some extent in the C-A mode. Figure 3 provides a suitable example for illustration. When using C-A single-channel recordings to measure inwardly rectifying K^+^ (Kir) channels, if the bath solution is normal Tyrode’s solution (containing ~5.4 mM K^+^) and the recording pipette is filled with a K^+^-rich solution (~140 mM), the reversal potential—that is, the potential at which single-channel current neither flows inward nor outward—becomes positive as a function of Δvoltage (Figure 3B). This occurs because the patch potential is the sum of the resting potential and the pipette potential, while Δvoltage is defined as the potential relative to the bath [10,11,12]. In contrast, under the W-C configuration, the resting membrane potential of the cell is approximately –70 mV (Figure 2).

Figure 3A shows Kir-channel activity recorded from RAW 264.7 osteoclast precursors, which is sensitive to suppression by mitoxantrone. An osteoclast is a large, multinucleated cell responsible for breaking down bone tissue through a process called bone resorption. Mitoxantrone (Novantrone^®^, 1,4-dihydroxy-5,8-bis[2-(2-hydroxyethylamino)ethylamino]anthracene-9,10-dione), a synthetic anthracenedione chemotherapeutic agent used in cancer treatment and multiple sclerosis [14], has been observed to suppress Kir channels [13]. Notably, the channel activity was observed in the C-A mode, when outward currents were recorded at a potential of +50 mV relative to the bath (pipette potential), which, from the perspective of the cell membrane, actually represent inward K^+^ current. This voltage (i.e., patch potential) corresponds to approximately 120 mV relative to the resting membrane potential (50 plus 70 mV). Of note, this form of single-channel current exhibited a relatively long mean open time, making it readily distinguishable from other ion channels present in the same cells [10,13,15].

Figure 3B illustrates the current-voltage (I-V) relationships of Kir channels in lipopolysaccharide-differentiated RAW 264.7 cells, both in the absence and presence of mitoxantrone. Mitoxantrone treatment did not significantly alter the single-channel conductance, as reflected by the unchanged linear slope of the I-V curve. Importantly, the recorded currents are negative, which correspond to inward currents from the cellular perspective—indicating that K^+^ ions move from the extracellular space into the cytoplasm, driven by their electrochemical gradient [13,15].

The Kir2.x subfamily has been identified as the predominant inward rectifier expressed in differentiated RAW 264.7 cells [15]. However, it remains unclear whether mitoxantrone-induced inhibition of Kir channels represents an intrinsic property of neurons or glial cells, including microglia [10,15]. This mechanism is of particular interest, as modulation of Kir-channel activity may hold therapeutic potential in multiple sclerosis [14,16]. Multiple sclerosis is a chronic autoimmune demyelinating disorder in which the immune system attacks the central nervous system, leading to damage of the protective myelin sheath surrounding axons and subsequent impairment of neuronal signaling [17].

## 4. Differences Between ATP-Sensitive K^+^ (K_ATP_) Channels and Large-Conductance Ca^2+^-Activated K^+^ (BK_Ca_) Channels in the C-A Configuration

**Figure 4:**
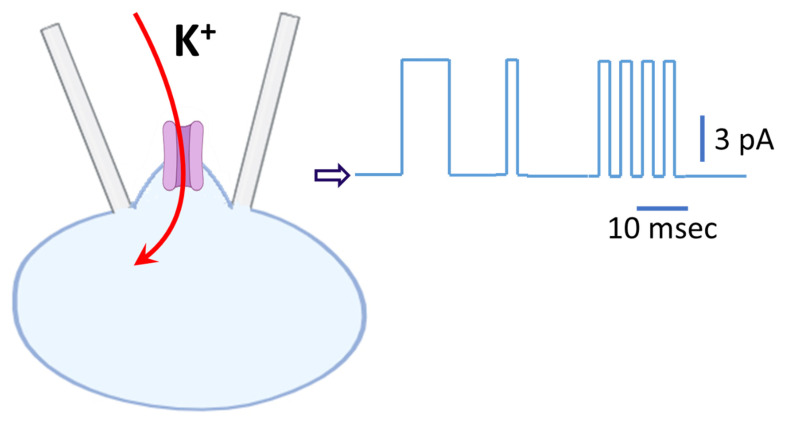
Schematic illustration of K_ATP_ channel activity in C-A configuration. The cell was bathed in standard Tyrode’s solution (~5.4 mM K^+^), while the recording pipette was filled with a K^+^-rich internal solution (~140 mM K^+^). The holding potential was set to −50 mV relative to the bath, corresponding to a driving force of −120 mV from the resting membrane potential (~+70 mV). Under these conditions, K^+^ ions enter the cell through K_ATP_ channel, driven by K^+^ electrochemical gradient, thereby producing an inward K^+^ current from the cell’s perspective (depicted by the red curved arrow). By convention, however, currents are defined relative to the recording pipette; thus, single-channel openings appear on the patch-clamp amplifier trace as random upward deflections, representing outward currents (illustrated on the right). Background noise superimposed on the single-channel trace was removed after idealization using the QUB package (http://qub.mandelics.com/, accessed on 26 January 2026). Calibration bars at the rightmost portion of the trace indicate current amplitude (vertical) and time (horizontal), with the open arrowhead marking the zero-current level.

When using the C-A single-channel recordings, the holding potential displayed on the instrument (i.e., patch amplifier) is negative, but for the cell being examined, it is actually positive. Therefore, what appears as a downward deflection representing inward current on the recording is, in fact, an upward deflection representing outward current from the cell’s perspective, as demonstrated in Figure 4. Because of this discrepancy, some early studies appeared to mistakenly report K_ATP_ channel activity when they were actually observing the activity of BK_Ca_ channels.

The large-conductance Ca^2+^-activated K^+^ channels—also known as BK, BK_Ca_, or maxi-K channels—are a unique type of channel. They open in response to membrane depolarization and elevated intracellular Ca^2+^, permitting large outward-rectifying K^+^ currents to flow [8,16,18,19]. The single-channel conductance of BK_Ca_ channels in symmetrical K^+^ solution (~140 mM) is 150–250 pS. However, when the bath solution contains low K^+^ (i.e., normal Tyrode’s solution), their conductance can be around 100–150 pS, a value which is close to that of K_ATP_ channel.

The ATP-sensitive K^+^ channels (K_ATP_ channels) are specialized K^+^ channels that couple a cell’s metabolic state to its electrical activity. When intracellular ATP levels are high, these channels close; when ATP levels decline, they open. Through this mechanism, K_ATP_ channels play a critical role in regulate physiological processes, such as hormonal release (i.e., insulin release), vascular tome, and cellular protection during metabolic stress [20].

Both of these channels exhibit mean open times on the order of a few milliseconds, whereas classical Kir channels (such as Kir2.1), as described above in RAW 264.7 cells, typically remain open for several hundred milliseconds (Figure 3), making them much easier to distinguish. It is thus important to note that K_ATP_ channels display bursting kinetics characterized by a mean intraburst open time of ~1 ms, whereas BK_Ca_ channels typically lack this behavior [19]. In addition, rectification properties provide an important means of distinguishing between the two: K_ATP_ channels display mild inward rectification, while BK_Ca_ channels exhibit strong outward rectification [16,18,19]. Figure 4 illustrates a schematic representation of K_ATP_ channel activity.

## 5. Interference of Single-Channel Activity by Action Currents (ACs) in C-A Current Recordings

When using C-A single-channel recordings, if the bath solution is normal Tyrode’s solution (containing ~5.4 mM K^+^), the recording electrode is filled with K^+^-rich solution, and the holding potential is very negative (e.g., below −80 mV), corresponding to a Δvoltage of about more than +10 mV relative to the bath, the appearance of inward currents observed from the pipette’s perspective may indicate the presence of BK_Ca_-channel currents. However, such currents could also be action currents (ACs), if the cell is highly electrically excitable. In this situation, the single-channel current traces are easily disturbed and distorted [5,6,8,9,19,20,21,22,23,24,25,26]. Consequently, analyzing the single-channel kinetics of BK_Ca_ channels (such as mean open time or mean closed time, derived from open-time or closed-time histograms) becomes severely compromised due to contamination by the concurrent activity of ACs. To minimize interference from ACs, one effective strategy is to replace the bath solution with a high-K^+^ solution (about 140 mM). This adjustment shifts the reversal potential for K^+^ ions toward 0 mV, thereby suppressing AC activity through prolonged depolarization of the cells. It needs to be noted, however, that prolonged exposure to high-K^+^ solution may diminish overall cellular activity.

When using C-A single-channel recordings in electrically excitable cells, ACs often appear (which correspond to the action potentials [APs] observed in W-C current-clamp recordings) [2,5,6,7,8,9]. Typically, their deflections in the same direction as those of BK_Ca_ channels, although their directions are biphasic to some extent. Moreover, the duration of ACs is relatively short, whereas the duration of BK_Ca_ channel openings can vary, sometimes brief and sometimes prolonged. However, when the activity of BK_Ca_ channels is relatively low, their opening duration is also short, making them indistinguishable from ACs.

In W-C recordings, when using the voltage-clamp configuration combined with variable voltage-clamp protocols enables the observation of many distinct types of macroscopic ionic currents. Switching to the current-clamp configuration with the holding current set to 0 pA allows for measurement of the cell’s the resting membrane potential. Furthermore, in electrically excitable cells, appropriate current injection can elicit action potentials (APs), and spontaneous APs may occasionally be observed [2,8,16]. Sustained AP firing can also occur when a constant holding current is applied, thereby depolarizing the membrane potential [6,8].

In the early days, commonly used microelectrode amplifiers such as the Axoclamp-2B (Molecular Devices, San Jose, CA, USA) made switching from single-electrode voltage-clamp mode to current-clamp mode rather cumbersome and difficult to operate. In contrast, many modern patch amplifiers—such as Axopatch 1D, Axopatch 200B (Molecular Devices), EPC-7 (HEKA Elektronik, Lambrecht, Germany), or RK-400 (Bio-Logic, Claix, France)—allow for much easier switching between voltage-clamp and current-clamp modes [2,8,12]. Nevertheless, the observed phenomena are a consequence of the patch-clamp method, independent of the specific amplifier model used.

In C-A voltage-clamp recordings, single-channel events are frequently detectable; however, action currents (ACs), which correspond to action potentials (APs) observed in whole cell current-clamp recordings, may also be recorded [8,9,12,24,25,27,28]. Moreover, a shift from C-A voltage-clamp to current-clamp recording mode often results in observable changes in membrane potential. Yet, under this circumstance, due to differences in the position of the holding current, the true resting membrane potential cannot be accurately measured, particular when the bath solution contains low K^+^ (approximately 5.4 mM K^+^). In addition, under C-A current-clamp mode, spontaneous APs can easily be confused with subthreshold depolarizations or synaptic potentials accompanied by spiking firing. Subthreshold oscillations refer to rhythmic fluctuations in a neuron’s membrane potential that occur below the threshold required to trigger an AP [7,8,24,26,27,28,29,30].

## 6. Modification of BK_Ca_-Channel Activity by GAL-021 in GH_3_ Cells

**Figure 5:**
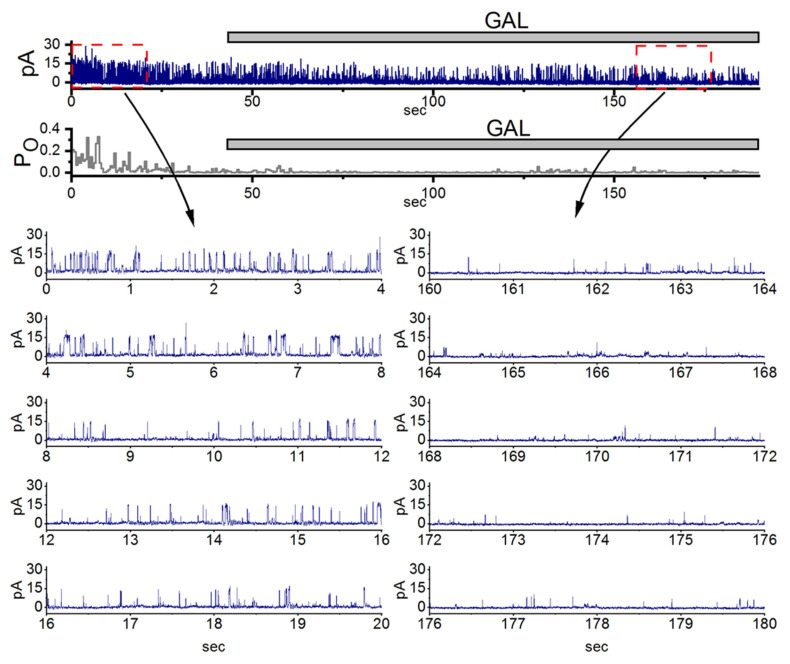
Inhibitory effect of GAL-021 on the activity of large-conductance Ca^2+^-activated (BK_Ca_) channels in pituitary GH_3_ cells. GH_3_ cells were bathed in a high K^+^ solution containing 0.1 μM Ca^2+^, and inside-out patch-clamp recordings were obtained at +60 mV (equivalent to −60 mV from the electrode’s perspective). In the uppermost part of the figure, the upper portion displays representative current trace, while the lower portion shows the corresponding channel opening probability. The horizontal bar indicates the application of 10 μM GAL-021 (GAL) into the bath solution. Expanded current traces in the lower panel (left, control; right, during GAL-021 exposure) correspond to the regions marked by red dashed boxes with black curved arrows in the uppermost panel. From the cell’s perspective, channel openings are represented as upward deflections (outward currents). Notably, exposure to GAL-21 (10 μM) progressively reduced the channel open probability. This figure is adapted from Lu et al. [18] and is published under the Creative Commons Attribution (CC BY) license.

In this study, we further demonstrated that inhibitory effect of GAL-021 on the channel opening of BK_Ca_ channels. The compound GAL-021 (also known as ENA-001, 6-(methoxy(methyl)amino)-N^2^,N^4^-di-propyl-1,3,5-triazine-2,4-diamine) is a small-molecule drug developed as a novel breathing control modulator and a respiratory stimulant. As demonstrated in Figure 5, under high-K^+^ conditions, the activities of these channels can be readily observed in an excised inside-out patch. Notably, in the inside-out configuration, the activity of ACs observed in C-A mode is absent. When the detached patch was exposed to GAL-021, the activity of channel openings was progressively reduced [18,31,32,33]. The open-state probability of the channel measured at the level of +60 mV under control condition (no GAL-021) was found to b1 0.182 ± 0.012 (*n* = 8). The addition of GAL-21 (10 μM) to the bath medium decreased the channel activity to 0.022 ± 0.002 (*n* = 8, *p* < 0.01). However, there was no significant difference in the amplitude of the unitary outward current between the absence and presence of GAL-021. It is clear that the presence of GAL-021 can decrease the opening probability of BK_Ca_ channels in these cells, but not that of K_ATP_ channels [19,33].

**Figure 6:**
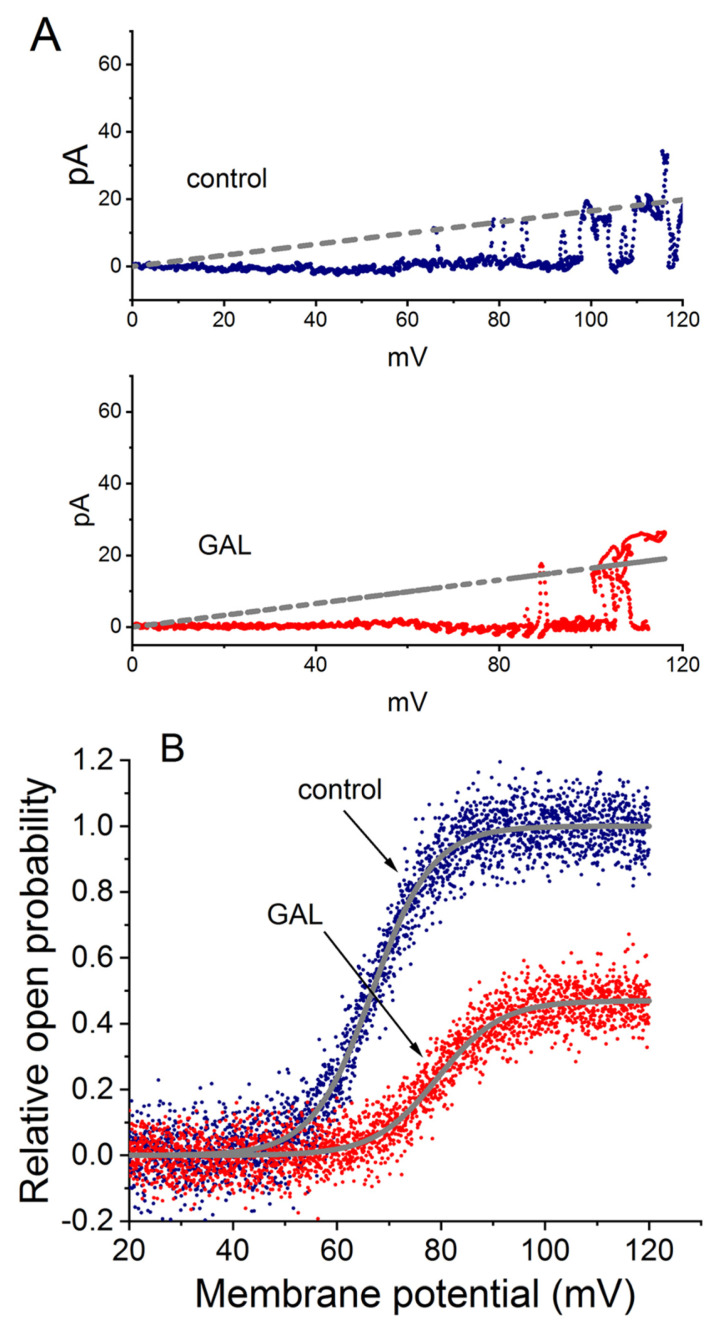
Voltage-dependent effects of GAL-021 (GAL) on BK_Ca_ channel activity in GH_3_ cells. Experiments were performed under symmetrical K^+^ concentration using K^+^-rich internal solution in inside-out patch recordings. The potential was held at +60 mV, and the bath medium contained 0.1 μM Ca^2+^. (**A**) GAL-021 did not alter the single-channel conductance of BK_Ca_ channels. Voltage ramp pulses (0 to +120 mV, 1 s duration) were applied to measure conductance in the absence or presence of 3 μM GAL-21. The gray straight dashed line (reversal potential of 0 mV) illustrates the I-V relationship, with traces shown for control (blue, upper) and GAL-021-treated (red, lower) conditions. (**B**) GAL-021 shifted the voltage-dependent activation of BK_Ca_ channels. Currents were evoked by ramp pulses from +20 to +120 mV (1 s duration). Boltzmann fits (gray smooth lines) yielded a half-activation voltage (V_1/2_) of +66.8 mV under control condition and +79.3 mV following exposure to 3 μM GAL-021. This figure is adapted from Lu et al. [18] and is published under the Creative Commons Attribution (CC BY) license.

The study also investigated the effect of GAL-021 on the single-channel conductance of BK_Ca_ channels using the voltage-clamp technique on inside-out patches. This method allowed for the construction of single-channel amplitude versus membrane potential (I-V) relationships by applying voltage ramp pulses from 0 to +120 mV for 1 s over 1 s. Figure 6A depicts the single-channel amplitude versus membrane potential (I-V) relationships of BK_Ca_ channels with or without the addition of GAL-021 (3 μM). The single-channel conductance of BK_Ca_ channels achieved from the linear I-V relationship in control (no GAL-021) was 165 ± 7 pS (*n* = 11) with a reversal potential of 0 ± 1 mV (*n* = 11). The value was not found to differ significantly from that (164 ± 6 pS; *n* = 11, *p* > 0.05) measured in the presence of GAL-021 (3 μM). Therefore, GAL-021 produced no significant change in the single-channel conductance of BK_Ca_ channels, but it suppressed the channel activity in these cells, along with a prolongation in mean closed time of the channel [18]. The results suggest a mechanism where GAL-021 stabilizes the channel in a non-conducting (closed) state without physically blocking the open pore.

Figure 6B illustrates the activation curve of BK_Ca_ channels taken from the absence or presence of GAL-021 (3 μM). To determine the effect of GAL-021 on the activation curve of BK_Ca_ channels, the upsloping ramp pulses from +20 to +120 mV with a duration of 1 s were designed and creased from pCLAMP 10.7, and they were then applied to the membrane patch through digital-to-analog conversion. During the single-channel current recordings, the linear ramp pulses were delivered from +20 to +120 mV for 1 s. The activation curve obtained during ramp pulses were calculated by averaging the current traces from 20 voltage ramps, then normalizing each mean current value to the corresponding single-channel amplitude at that potential, after correcting for the leakage component. The plot of the relative open probability as a function of membrane potential applied were thereafter constructed and then fitted with a modified Boltzmann function. In control, V_1/2_ = +66.8 ± 2.3 mV and *q* = 4.3 ± 0.2 *e* (*n* = 7), whereas in the presence of GAL-021 (3 μM), V_1/2_ = +79.3 ± 2.6 mV and *q* = 4.1 ± 0.2 *e* (*n* = 7). Consequently, the presence of GAL-021 (3 μM) caused a 45% decrease in the maximal open probability of BK_Ca_ channels. However, there was no significant effects on the gating charge of the curve occurring during exposure to GAL-021. This study led us to indicate that GAL-021 could suppress the activity of these BK_Ca_ channels in a voltage-dependent fashion in GH_3_ cells [18]. GAL-021 may possess a distinctive structural property that enables functional interaction with the a-subunit of the BK_Ca_ (*KCNMA1*) channel, thereby reducing the amplitude of whole-cell Ca^2+^-activated K^+^ currents [18].

## 7. Brain Slice Patch-Clamp Recordings

**Figure 7:**
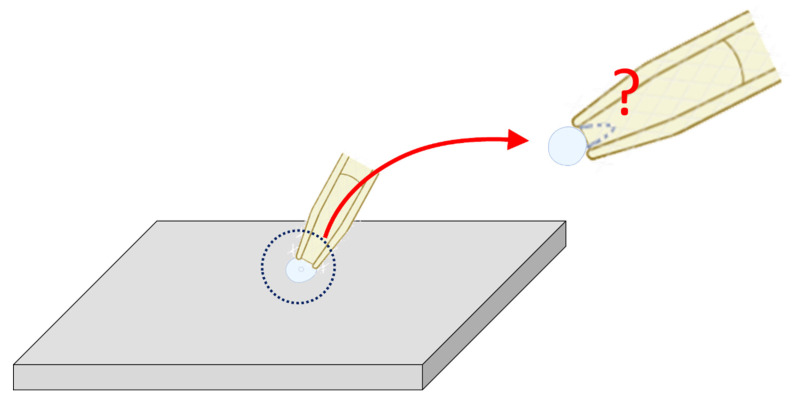
Schematic illustration of patch-clamp recording in brain slices. This diagram highlights a challenge encountered when performing patch-clamp recording on small neurons (<1 μm in diameter) within brain slices. In such cases, distinguishing between C-A and W-C configurations can be difficult, particularly when membrane capacitance changes are negligible after rupturing the patch membrane. A gray, flat three-dimensional parallelogram block represents the brain slice; notably, the depicted neuron is smaller than those in Figure 1. The top-right inset provides an enlarged view of the central dotted circle, emphasized by a red curved arrow. A red question mark denotes the uncertainty in determining whether the patch membrane remains intact or has ruptured, with the ruptured state indicated by a blue dashed curve.

A brain slice is a thin section of brain tissue, usually prepared with a vibratome, that is kept alive in artificial cerebrospinal fluid. Researchers use brain slices because they preserve much of the brain’s local circuitry while allowing for direct experimental access [9,33,34]. Brain slice patch-clamp recording is a commonly used electrophysiological method. However, because the patch electrode has a relatively large tip diameter, only sufficiently large neurons can be recorded appropriately. In addition, since the cell membrane is very fragile, it is often difficult during the procedure to distinguish whether the recording configuration in W-C mode or C-A mode, as demonstrated in Figure 7. When the electrode tip is too small (e.g., less than 1 μm), the membrane may reseal after membrane rupture in W-C mode, thereby reverting a C-A configuration. Furthermore, interference from excitatory or inhibitory synaptic currents adds to the complexity [2,7,9,12,24,25,26,27,32,33,34,35]. As a result, interpretation of the recordings becomes more challenging, and the reproducibility of experimental outcomes is greatly affected [2,7,24,32,34]. Therefore, in experiments such as brain slice patch clamping, the size of the cell is an important factor for the operator to consider when selecting cells for electrophysiological measurements (Figure 7).

## 8. Conclusions

In the cell-attached (C-A) configuration, many highly excitable cells readily display spontaneous or stimulation-evoked action currents (ACs) [5,8,9,25,26,27,28]. These ACs often correspond to the action potential firing observed under the whole-cell (W-C) configuration. In addition, the C-A mode allows for the detection of single ion channel activity in various cells. However, channels such as large-conductance Ca^2+^-activated K^+^ (BK_Ca_) channels are influenced by the presence of ACs, which complicates the analysis of their kinetics [6,7,21,22]. Finally, it should to be noted that in brain slice patch-clamp recordings, when the target cells are very small, distinguishing between W-C and C-A modes becomes difficult. Consequently, the assessment of AC activity is also challenging [26,28,31,32,33].

In earlier electrophysiological experiments, amplifiers were typically connected to oscilloscopes to observe the dynamic changes in voltage and current. Subsequently, through a data acquisition board, analog-to-digital conversion was performed to transform analog signals into digital form, allowing for effective storage on personal computers [2,8,36]. Nowadays, digital-to-analog conversion capability has also been incorporated, enabling various digitized voltage profiles—such as specific ramp voltage shapes or realistic action potential waveforms—to be reconverted into analog signals. These analog signals are subsequently employed to precisely regulate the patch amplifier and, in turn, the electrical environment of the cells, as described from the pCLAMP^TM^ 11 software suite or the HEKA system (https://www.heka.com/). Moreover, while strip chart recorders were once used to print experimental results, personal computers are now employed for analysis and output.

As a result, these developments have made it difficult for senior electrophysiologists to update or replace their instruments or devices in line with advances in technology and artificial intelligence. Meanwhile, younger scholars tend to focus more on computer operation, often neglecting the practical skills and techniques of patch-clamp recording. Additionally, automated patch-clamp system (such as QPatch^®^; Sophion Bioscience; Ballerup, Denmark, and the Port-a-Patch^®^ system; Nanion Technologies GmbH; Munich, Germany), as well as HEKA’s manual patch-clamp amplifier (HEKA Technologies GmbH), has led to significant breakthroughs in the study of various voltage-gated and ligand-gated ion currents. Although automated patch clamp systems efficiently replace manual methods for isolated cells, brain slice patch-clamp recordings remain a manual technique. Effective robotic platforms used for complex tissue slices awaits further technological developments [28,31,33,34,37,38,39,40]. For example, artificial intelligence (AI)-driven image recognition can identify suitable cells for recordings under the microscope. Machine learning algorithms can guide micropipette positioning with high precision, reducing human error. It is hoped that AI can help determine when to transition from C-A to W-C mode by analyzing resistance and capacitance changes automatically in the near future. Nevertheless, the scientific findings rather than technical background need to emphasized.

## Data Availability

No new data were created or analyzed in this study. Data sharing is not applicable to this article.

## Abbreviations

AC	action current
AP	action potential
BK_Ca_	channel, large-conductance Ca^2+^-activated K^+^ channel
C-A	cell-attached
I-V	current versus voltage
Kir channel	inwardly rectifying K^+^ channel
K_ATP_ channel	ATP-sensitive K^+^ channel
W-C	whole-cell
